# Retraction Note: The role of social circle COVID-19 illness and vaccination experiences in COVID-19 vaccination decisions: an online survey of the United States population

**DOI:** 10.1186/s12879-023-08234-8

**Published:** 2023-04-11

**Authors:** Mark Skidmore

**Affiliations:** Department of Agricultural, Food and Resource Economics, Department of Economics, Michigan State University, 91 Morrill Hall of Agriculture, East Lansing, MI 48824–1039 China

**Retraction Note: BMC Infectious Diseases (2023) 23:51**

10.1186/s12879-023-07998-3

The editors have retracted this article as concerns were raised regarding the validity of the conclusions drawn after publication. Post-publication peer review concluded that the methodology was inappropriate as it does not prove causal inference of mortality, and limitations of the study were not adequately described. Furthermore, there was no attempt to validate reported fatalities, and there are critical issues in the representativeness of the study population and the accuracy of data collection. Lastly, contrary to the statement in the article, the documentation provided by the author confirms that the study was exempt from ethics approval and therefore was not approved by the IRB of the Michigan State University Human Research Protection Program.

The author disagrees with this retraction.

